# How to write a case report in nephrology

**DOI:** 10.1186/s13256-016-0852-4

**Published:** 2016-04-06

**Authors:** Varun Agrawal

**Affiliations:** Division of Nephrology and Hypertension, University of Vermont College of Medicine, 1 South Prospect Street, Burlington, VT 05401 USA

## Abstract

Nephrologists provide medical care to patients presenting with acute kidney injury, chronic kidney disease, glomerular diseases, and electrolyte or acid–base disorders, and perform lifesaving dialysis therapies and kidney transplantation. This editorial is an attempt to describe how to write a comprehensive, yet succinct, case report in nephrology. The essential elements of a nephrology case report are defined that can serve as a writing aid to the author.

Nephrology is an exciting medical subspecialty that deals with study of the myriad ways that the kidney strives to maintain internal homeostasis. Abnormalities of kidney function thus lead to derangements in fluid, electrolyte and acid–base status. Nephrologists deal with a variety of disorders in the in-patient and out-patient settings that make it a challenging as well as a rewarding experience.

Many case reports in nephrology are about acute kidney injury (sometimes needing dialysis), acute glomerulonephritis requiring kidney biopsy or immunosuppression, electrolyte disorders or interesting radiological findings. While general principles on writing a case report have been reviewed elsewhere [1], in this editorial I offer guidelines (Table 1) on how to effectively write a case report in nephrology. Along with the CARE (CAse REport) guidelines for standardized reporting [2, 3], these suggestions can dramatically shorten the time taken to adjudicate a case report and may even enhance the chances of acceptance of the author’s work.

**Table 1:** Essential elements of a nephrology case report

Acute kidney injury:
● Serum creatinine (at presentation and at baseline), estimated glomerular filtration rate
● 24-hour urine output (if able to quantify)
● Urine analysis (especially blood and protein in urine)
● Urine microscopy
● Imaging of kidneys (if available)
● Medication review to evaluate for nephrotoxic medications
Acute glomerulonephritis/nephrotic syndrome:
● Hypertension
● Systemic symptoms/signs (edema, anasarca, arthritis or rash)
● Urine analysis (especially blood and protein in urine)
● Urine microscopy (specifically presence or absence of dysmorphic red blood cells or casts)
● Urine albumin to creatinine ratio (urine protein to creatinine ratio or 24-hour urine protein)
● Kidney biopsy findings
Kidney biopsy:
Light microscopy
● Number of glomeruli obtained in biopsy sample
● Proportion of glomeruli affected
● Description of pathological findings (with hematoxylin and eosin or periodic acid–Schiff staining) and any special staining
Immunofluorescent microscopy
● Type of immunoglobulin and complement evaluated
● Staining pattern (location) and degree (trace to 3+)
Electron microscopy
● Significant findings (helpful if images can be included)
Drug therapies in glomerular disorders:
● Specify details on immunosuppression regimen for induction and maintenance, mode of administration (oral or intravenous), dose and frequency
● Include details on evidence of remission or lack of response
Dialysis therapy:
● State type of dialysis (intermittent hemodialysis, continuous renal replacement therapy or peritoneal dialysis)
● Type of dialysis access
● Dialysis prescription (frequency, duration, composition and volume of dialysate fluid) and any significant machine settings
Electrolyte and acid–base disorder:
● Details of electrolyte disorder at baseline and at presentation
● Appropriate blood and urine tests done to determine cause of electrolyte disorder
● Treatment offered and if this led to correction of the electrolyte disorder
Imaging finding:
● Type of study
● Specialized imaging (with or without contrast) and images of the radiological studies: ultrasound, computed tomography (CT) or magnetic resonance imaging (MRI)
Kidney transplant recipient:
● State reason for kidney failure
● Type of kidney transplant (deceased or living donor)
● Human leukocyte antigen matching
● Timing of transplantation
● Any prior episodes of rejection
● Prescribed immunosuppressants
Kidney stone:
● Presenting symptoms
● Imaging studies
● Study of stone composition
● 24-hour urine studies (if any)
● Treatment offered (urological intervention or stone prophylaxis)

If an element is missing or unavailable, please state the absence of the data as a limitation. This practice assures the editor and reader that every possible attempt was made to obtain the piece of information and demonstrates appropriate thought processes

## Description of case reports in nephrology

Acute kidney injury is the preferred term as opposed to acute renal failure or dysfunction. Serum creatinine levels at presentation and at baseline are necessary elements. Besides the usual workup and a careful medication review, the most likely cause of acute kidney injury should be clearly stated in a case report. Acute glomerulonephritis is an important cause of acute kidney injury that is reversible in many cases. The diagnosis of acute glomerulonephritis is confirmed *only* with a kidney biopsy. Thus, kidney biopsy findings are essential in a case report on acute glomerulonephritis.

A clear description of the pathological findings with images (at least images of light microscopy) is very important to give an exact idea of the type and severity of glomerular damage. This is also important as it allows other investigators to cite the case report and include details of the kidney biopsy in systematic reviews. Immunosuppressive therapy needs to be clearly described (frequency, dose and drug formulation) to allow other clinicians to benefit from this information. Further workup including specialized tests (such as activity of alternate complement pathway, serum cryoglobulins, serum free light chains) or genetic studies help solidify the diagnosis and are highly regarded when available.

Many electrolyte disorders are due to changes in intake, output (especially through the kidneys or gastrointestinal tract) or transcellular shift. In a case report where the electrolyte disorder is diagnosed to be the result of disturbance in function of the renal tubules, a thorough attempt should be made at eliminating every other possibility. Usually, urine studies (such as urine potassium concentration in hypokalemia, urine magnesium concentration in hypomagnesemia, urine pH in renal tubular acidosis) need to be described if the kidney is contributing to the electrolyte disorder. Abnormalities on renal imaging require high-resolution images to best demonstrate the radiological abnormality, and if any confirmatory testing (such as genetic testing for polycystic kidney disease or tuberous sclerosis) was performed then it should be described.

In conclusion, a thorough understanding of renal pathophysiology is essential to explain what abnormality leads to a particular problem pertaining to the kidney and what diagnostic tests and therapeutic options can be offered to the patient. Accuracy, brevity, and clarity go a long way in ensuring that a case report in nephrology offers an exceptional learning opportunity to physicians in this exciting field.
